# Case Series: *ATRX* Variants in Four Patients with Metastatic Pheochromocytoma

**DOI:** 10.3389/fendo.2024.1399847

**Published:** 2024-09-16

**Authors:** Briana N. Cortez, Mickey J. M. Kuo, Abhishek Jha, Mayank Patel, Jorge A. Carrasquillo, Tamara Prodanov, Kailah M. Charles, Sara Talvacchio, Alberta Derkyi, Frank I. Lin, David Taïeb, Jaydira Del Rivero, Karel Pacak

**Affiliations:** ^1^ Section on Medical Neuroendocrinology, Eunice Kennedy Shriver National Institute of Child Health and Human Development, National Institutes of Health, Bethesda, MD, United States; ^2^ Medical Genetics Branch, National Human Genome Research Institute, National Institutes of Health, Bethesda, MD, United States; ^3^ Center for Cancer Research, Laboratory of Pathology, National Cancer Institute, Bethesda, MD, United States; ^4^ Molecular Imaging Branch, National Cancer Institute, National Institutes of Health, Bethesda, MD, United States; ^5^ Department of Nuclear Medicine, La Timone University Hospital & Centre de Recherches en Cancérologie de Marseille (CERIMED) & French Institute of Health and Medical Research (Inserm) UMR1068 Marseille Cancerology Research Center, Institut Paoli-Calmettes, Aix-Marseille University, Marseille, France; ^6^ Developemental Therapeutics Branch, National Cancer Institute, National Institutes of Health, Bethesda, MD, United States

**Keywords:** case report, pheochromocytoma, ATRX, prognosis, imaging

## Abstract

Few reports have highlighted the rare presence of somatic *ATRX* variants in clinically aggressive, metastatic pheochromocytoma/paraganglioma (PCC/PGL); however, none have addressed detailed clinical presentation (including biochemistry and imaging) and management of these patients. Here, we address these clinical features and management based on four PCC patients with somatic *ATRX* variants from our National Institutes of Health PCC/PGL cohort. A total of 192 patients underwent exome sequencing (germline, somatic, or both), and four males were found to have somatic *ATRX* variants (with additional somatic *VHL* and *FH* oncogenic variants in patients 2 and 4, respectively). Per-lesion and per-patient comparisons were performed among functional imaging scans performed at the NIH. Biochemical phenotype and response to systemic treatment were evaluated. This mini-series supports prior studies showing aggressive/metastatic PCC in patients with somatic *ATRX* variants, as all developed widespread metastatic disease. All four PCC patients presented with noradrenergic biochemical phenotype, and some with significant elevation in 3-methoxytyramine. ^18^F-FDOPA PET/CT was found to be the superior functional imaging modality, with 100% lesion detection rate when compared to that of ^68^Ga-DOTATATE, ^18^F-FDG, ^18^F-FDA, and ^123^I-MIBG scans. While patients did not respond to chemotherapy or tyrosine kinase inhibitors, they responded to targeted radiotherapy using high-specific-activity ^131^I-MIBG (Azedra^®^) or ^177^Lu-DOTATATE (Lutathera^®^).

## Introduction

Pheochromocytomas and paragangliomas (PCCs/PGLs) are neuroendocrine tumors derived from neural crest cells. They produce, metabolize, and secrete catecholamines and their metabolites, metanephrines and 3-methoxytyramine. While PCCs originate from sympathetic paraganglia of the adrenal medulla, PGLs arise from extra-adrenal autonomic nervous system ganglia and their accompanying nerves (1). In 2017, the World Health Organization Classification of Tumors of Endocrine Organs identified all PCC/PGLs as having metastatic potential based on similar histology, thus eliminating the use of “benign” and “malignant” (1).

In PCCs/PGLs, abnormally elevated plasma 3-methoxytyramine levels, large tumor size (mostly over 5–6 cm), and extra-adrenal location all significantly correlate with metastatic disease and consequently with poor prognosis (2–4). Furthermore, pathogenic variants in genes encoding succinate dehydrogenase complex A (*SDHA*) and B (*SDHB*), and endothelial PAS domain protein 1/hypoxia-inducible transcription factor 2α (*EPAS1/HIF2A*) correlate with increased risk of metastasis (5–9). Other risk factors include TERT overexpression, pS100 loss, presence of brown adipose tissue, tumor necrosis, and vascular invasion (10–14). Small studies have shown that somatic *ATRX* pathogenic variants also correlate with aggressive/metastatic PCC/PGL (7, 15).

Germline *ATRX* pathogenic variants are associated with the rare neurodevelopmental disorder alpha-thalassemia X-linked intellectual disability syndrome (16). More recently, the role of ATRX in tumor suppression has been described. Somatic *ATRX* pathogenic variants correlate with initiation and progression of tumors, including pancreatic neuroendocrine tumors, neuroblastoma, and pediatric osteosarcoma (17–20). ATRX maintains chromatin remodeling and telomere maintenance, essential in tumorigenesis and replicative immortality. Telomeres are maintained by telomerase or a telomerase-independent mechanism involving homologous recombination, named alternative lengthening of telomeres (ALT) (21). ATRX and the death domain associated protein (DAXX), work jointly to maintain telomere chromatin structure and replication by adding histone H3.3 into heterochromatin. Telomere stress develops in the absence of ATRX, leading to ALT (22). A pioneering study by Fishbein et al. was the first to report the presence of *ATRX* variants in clinically aggressive PCC/PGLs. In their discovery and validation cohorts, 9.5% and 12.6% of PCC/PGL samples had somatic *ATRX* pathogenic variants, respectively (23). Use of telomere fluorescence *in situ* hybridization (FISH) showed ALT in the presence of *ATRX* variants. Another study by Job et al. found somatic *ATRX* variants in 4.8% of their PCC/PGL cohort, which were most frequently seen in the setting of germline *SDHB* pathogenic variants. Additionally, out of 25 PCC/PGL patients with ALT in their study, 24% had *ATRX* variants with poorer prognosis and greater metastatic risk (24).

Thus, in this present study, we focused on PCC/PGL patients enrolled in our National Institutes of Health (NIH) IRB-approved protocol (NCT00004847) with somatic *ATRX* loss-of-function variants to describe their comprehensive evaluation, including biochemistry, genetics, functional imaging, and clinical outcomes.

## Case presentations

### Patient 1

A male patient with PCC passed at age 71, 11 years after his initial diagnosis. He initially presented in 1999 with diaphoresis, palpitations, left-sided varicocele, and uncontrolled hypertension (HTN) despite pharmacological intervention. An abdominal ultrasound and subsequent computed tomography (CT) scan found a 14 cm left adrenal tumor. Subsequent biochemical testing revealed elevated plasma norepinephrine (2850 pg/mL [<175 pg/mL]). He underwent a left adrenalectomy and nephrectomy. Histopathology reported a well-encapsulated PCC with no invasion into the kidney or surrounding structures. Three years later, he experienced recurrent night sweats, irritable bowel symptoms, and HTN (up to 170/110 mmHg), with elevated plasma normetanephrine (681 pg/mL [<175 pg/mL]) and chromogranin A (CgA) (244 ng/mL [<76 ng/mL]). CT, ^18^F-fluorodeoxyglucose (^18^F-FDG) positron emission tomography (PET)/CT, ^123^I-metaiodobenzylguanidine (^123^I-MIBG) scintigraphy, and technetium-99m methylene diphosphonate (MDP) bone scintigraphy detected multiple metastatic lesions, including lungs, lymph nodes, and bones. Due to ^123^I-MIBG-avid metastatic lesions, he began ^131^I-MIBG radiotherapy with interval improvement in periaortic and multiple abdominal lymph nodes. The following year, widespread metastatic progression was noted, involving the lungs, lymph nodes, and bones. He was referred to our team at the NIH and underwent ^18^F-fluorodopa (^18^F-FDOPA) PET/CT, ^18^F-fluorodopamine (^18^F-FDA) PET, and anatomic imaging, which confirmed extent of metastatic involvement. Upon our recommendations, he underwent a right middle lobectomy and mediastinal node dissection. Plasma normetanephrine (313 pg/mL [18–122 pg/mL]) and CgA (635 ng/mL [<225 ng/mL]) remained elevated. Due to progressive and extensive metastases, he began treatment with cyclophosphamide, vincristine, and dacarbazine (CVD). He completed 3 cycles, complicated by neutropenia and peripheral neuropathy. Further progression of the disease was observed in the lungs, lymph nodes, and bone, leading to the discontinuation of CVD therapy. He planned to receive radiation therapy to a growing right hilar mass in his home state; however, he was lost to follow-up and the NIH was notified of the patient’s death 2 years later from extensive metastatic disease.

### Patient 2

**Figure 1:**
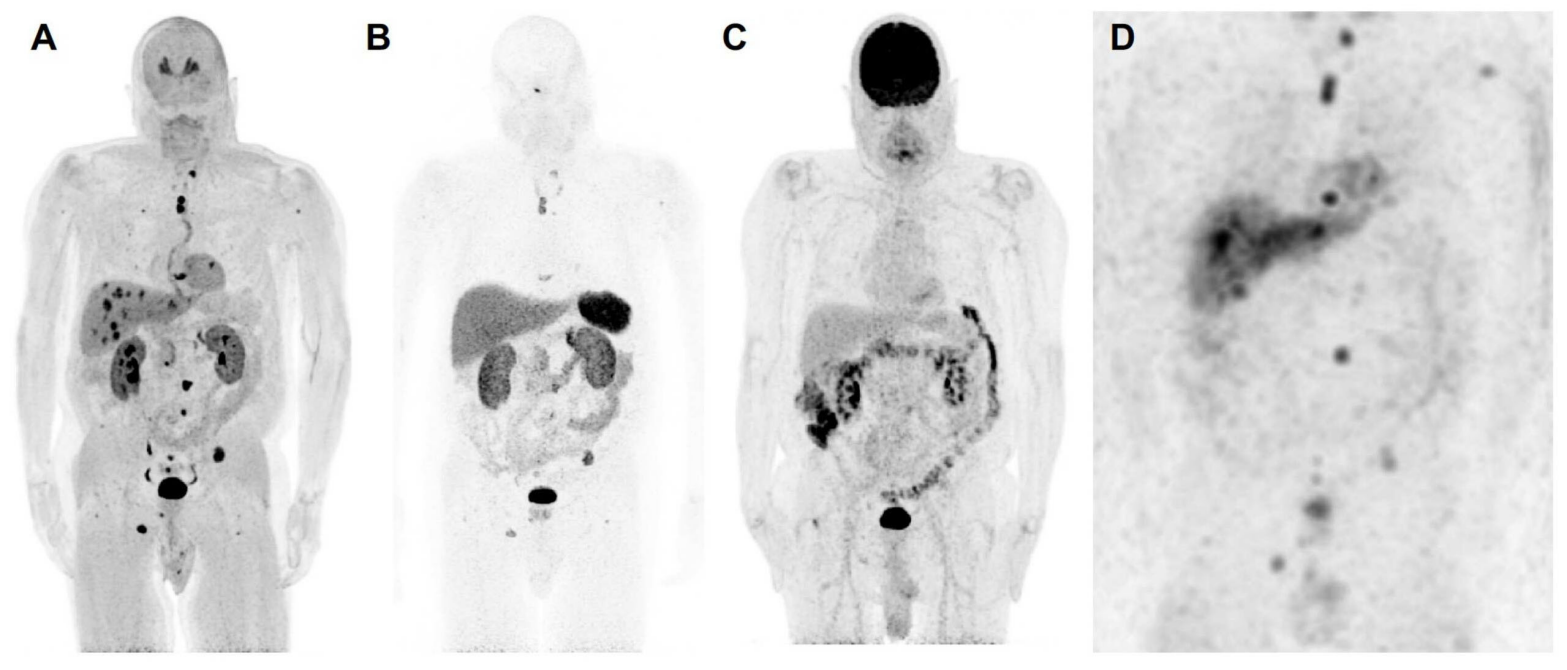
The anterior maximum intensity projection images of ^18^F-FDOPA positron emission tomography/computed tomography (PET/CT) **(A)**, ^68^Ga-DOTATATE PET/CT **(B)**, ^18^F-FDG PET/CT **(C)**, and ^123^I-MIBG single photon emission computed tomography/computed tomography (SPECT/CT) **(D)** of a 67-year-old male (patient 3) with a likely oncogenic somatic *ATRX* variant (c.2018dup, p.Thr674fs) with a history of a previously resected 8.7 cm right pheochromocytoma demonstrates metastatic lesions in lungs, liver, and bones. ^18^F-FDOPA PET/CT shows superiority in detection of metastatic lesions compared to ^68^Ga-DOTATATE PET/CT, ^18^F-FDG PET/CT, and ^123^I-MIBG SPECT/CT. Liver lesions are detected by ^18^F-FDOPA PET/CT and ^123^I-MIBG SPECT/CT and are not seen on ^68^Ga-DOTATATE PET/CT and ^18^F-FDG PET/CT. Furthermore, ^18^F-FDOPA PET/CT detects more bone and lung lesions compared to ^68^Ga-DOTATATE PET/CT, ^18^F-FDG PET/CT, and ^123^I-MIBG SPECT scintigraphy. Based on widespread avidity of metastatic lesions on ^123^I-MIBG scintigraphy compared to ^68^Ga-DOTATATE PET/CT, the patient was recommended for high-specific-activity ^131^I-MIBG (Azedra^®^) targeted radiotherapy. Additionally, ^18^F-FDG PET/CT demonstrates diffuse increased uptake in the colon, likely physiologic and not suggestive of malignancy. The ^18^F-FDA PET/CT, unfortunately, was not performed in this patient. To note, contrast recovery, sensitivity, and spatial resolution of PET/CT imaging is superior to SPECT/CT imaging, and therefore, smaller lesions on PET/CT scans may not be visible on SPECT/CT scans despite adequate uptake.

A 28-year-old male initially presented to the emergency room with a pounding headache, abdominal pain, and nausea in 1987 and was admitted to the hospital with hypertensive crisis. Magnetic resonance imaging (MRI) identified a right adrenal mass, for which he underwent a right adrenalectomy. Histopathology reported a PCC measuring 13 cm. He was lost to follow-up, and twenty-six years later, MRI, CT, ^18^F-FDG, ^18^F-FDOPA, and ^68^Ga-DOTA(0)-Tyr(3)-octreotate (^68^Ga-DOTATATE) PET/CT, and ^123^I-MIBG scintigraphy revealed multiple tumors in the abdomen, lungs, bones, and an aortocaval mass. Biochemical evaluation showed elevated plasma normetanephrine (1462 pg/mL [18–122 pg/mL]), dopamine (25 pg/mL [0–24 pg/mL]), and CgA (1349 ng/mL [<93 ng/mL]). He subsequently underwent an exploratory laparotomy, open cholecystectomy, and resection of the right aortocaval mass. The following year, he developed recurrence in the right adrenalectomy bed, and progression of metastatic lesions in the lungs, lymph nodes, and bones. One year later, he underwent a resection of a C2 paraspinal mass (**Figure 1**) and intensity-modulated radiation therapy (IMRT, 5400 cGy) to the right sacral area with shrinkage of the lesion. Due to continued progression, he began Sandostatin^®^ long-acting release (LAR) for 4 months, followed by sunitinib for 5 months. Sunitinib was discontinued due to uncontrolled HTN, flushing, ageusia, and weight loss. Due to progressive disease and recurrence of his C2 paraspinal mass, he was evaluated at the NIH and enrolled in our NCT03206060 protocol: Lu-177-DOTATATE (Lutathera^®^) in Therapy of Inoperable Pheochromocytoma/Paraganglioma Clinical Trial. He completed 4 cycles of treatment without progression and shrinkage in several lung, liver, and lymph node metastatic lesions. After 2 years, his CgA levels began to rise, and after 3 years, pulmonary progression was seen on CT imaging. He restarted ^177^Lu-Dotatate and completed 4 additional cycles. Since then, disease remains stable on imaging, and he remains in close follow-up with the NIH.

### Patient 3

A male patient with PCC passed at age 68, two years after his initial diagnosis. He initially presented with longstanding HTN, right back pain, pounding headaches, vertigo, fatigue, and multiple subcutaneous tumors. During routine abdominal ultrasound for risk of abdominal aortic aneurysm due to cigarette smoking, an incidental 1.7 x 1.6 x 1.4 cm hypoechoic lesion in the left liver lobe was identified, along with an enlarged paraaortic lymph node. He was evaluated at the NIH with anatomic and functional imaging scans confirming these lesions, along with a large 8.7 x 7 cm right adrenal mass, multiple masses in the liver, lungs, bone, and enlarged mesenteric and retroperitoneal lymph nodes. Histopathology from a liver biopsy specimen showed a well-differentiated neuroendocrine neoplasm, consistent with metastatic PCC. Biochemical evaluation showed elevated plasma normetanephrine (8592 pg/mL [<148 pg/mL]) and metanephrine (1261 pg/mL [<57 pg/mL]). Subsequently, he underwent a laparoscopic right adrenalectomy, with histopathologically confirmed PCC and Ki-67 < 3%. The following year, increasing size and number of metastatic lesions were noted in the lungs, liver, lymph nodes, and bones. Based on the avidity of his metastatic lesions on ^123^I-MIBG scintigraphy, he planned to start treatment with high-dose iobenguane-^131^I-MIBG (Azedra^®^). Approximately one year later, he was admitted to the emergency department experiencing shortness of breath and ultimately passed away due to complications from pneumonia and sepsis. It is believed that his advanced pulmonary metastatic disease played a role in his death.

### Patient 4

A male patient with PCC passed at age 64, twenty years after diagnosis. The patient initially presented with progressive HTN, palpitations, flushing, and uncontrolled type 2 diabetes mellitus. He was found to have an elevated vanillylmandelic acid; metanephrines were unavailable. He underwent an abdominal ultrasound and CT scan showing a large left-sided adrenal mass. Due to suspicion of PCC, he underwent a left radical adrenalectomy and nephrectomy without evidence of recurrence until 9 years later. At that time, he developed progressive HTN and uncontrolled hyperglycemia. He was evaluated at the NIH with MRI, CT, ^18^F-FDG, and ^123^I-MIBG scintigraphy, which revealed pulmonary, liver, lymph node, peritoneal, and left mesocolon metastasis and recurrence in the left surgical adrenal bed. Biochemical analysis revealed elevated plasma normetanephrine (3307 pg/mL [18–122 pg/mL]), norepinephrine (4949 pg/mL [80–498 pg/mL]), dopamine (59 pg/mL [3–46 pg/mL]), and CgA (1940 ng/mL [≤ 225 ng/mL]). He subsequently underwent resection of left adrenal surgical bed recurrence and metastatic lesions located in the subdiaphragmatic lymph node and mesocolon. Three years later, he presented with worsening HTN and glucose management. Imaging detected pulmonary, liver, lymph node, and bone metastatic lesions. He subsequently underwent exploratory laparotomy with lysis of adhesions and resections of the left periaortic and retropancreatic lymph node masses. Approximately 3 years later, due to progressive disease, he completed 4 cycles of ^177^Lu-DOTATATE, followed by 14 months of lanreotide injections, and subsequently 3 cycles of CVD, all without stabilization of disease. The patient ultimately succumbed to metastatic disease.

## Diagnostic assessment and methods

### Patient selection and genetic testing

The study protocol was approved by the Intramural Program of the National Institutes of Health, the *Eunice Kennedy Shriver* National Institute of Child Health and Human Development Institutional Review Board (NCT00004847). Written informed consent was obtained for all clinical, genetic, biochemical, and imaging studies. Our institution complies with all applicable laws, regulations, and policies concerning privacy and confidentiality.

Among NIH study participants without known genetic etiology for a personal history of PCC/PGL, 192 patients underwent exome sequencing (germline, somatic, or both) through the Center for Cancer Research Sequencing Facility. Next-generation sequencing was run on a NovaSeq 6000 S2 using Agilent SureSelect Human All Exon V7 in paired-end sequencing mode. DRAGEN was used for mapping to the reference genome hg38, and variant calling. Germline and somatic variants in published PCC/PGL-associated genes (including *SDHA, SDHB, SDHC, SDHD, SDHAF2, VHL, RET, NF1, IDH1, KIF1B, HRAS, EPAS1, EGLN1, EGLN2, MAX, TMEM127, FH, BAP1, MDH2, ATRX, DLST, ACO1/IRP1, MAML3, GOT2*, and *DNMT3A*) identified by next-generation sequencing (NGS) were confirmed by targeted Sanger sequencing. After removal of false positive results (those not confirmed by Sanger sequencing) and variants classified as benign or likely benign, four males had somatic putative loss-of-function *ATRX* variants, including three variants that were classified as likely oncogenic and one as a variant of uncertain significance (VUS), as determined by application of the Standards for the Classification of Pathogenicity of Somatic Variants in Cancer (Oncogenicity) (25) (**Table 1**). Each of the four patients described in detail here underwent paired exome sequencing (i.e., germline and somatic sequencing with only one tumor used for each patient), and none were found to have germline pathogenic or likely pathogenic variants, nor any suspicious variants of uncertain significance in the genes analyzed. There were no other relevant somatic variants in the genes analyzed that passed confirmation by Sanger sequencing except for those listed explicitly in the Results section. Rare Exome Variant Ensemble Learner (REVEL) scores are derived from the aggregate of multiple computational tools to predict pathogenicity of missense variants as a probability, ranging from 0 to 1 (26).

**Table 1:** Somatic variants identified by exome sequencing of patient tumors.

Patient ID	Tissue	Germline or Somatic	Gene	Variant (c.)	Variant (p.)	ATRX Protein Domain	Exon	Variant Classification
1	Right middle lobe pulmonary nodule	Somatic	*ATRX*	c.5248C>G	p.Pro1750Ala	SNF2	20 of 35	Likely oncogenic*
2	Right retrocaval mass	Somatic	*ATRX*	c.3230del	p.Ser1077del	None	9 of 35	Likely oncogenic
		Somatic	*VHL*	c.505C>G	p.Leu169Val		3 of 3	Likely oncogenic
3	Right adrenal PCC	Somatic	*ATRX*	c.2018dup	p.Thr674fs	RBR	9 of 35	Likely oncogenic
4	Left adrenal surgical bed mass	Somatic	*ATRX*	c.5229G>T	p.Arg1743Ser	SNF2	20 of 35	Uncertain significance
		Somatic	*FH*	c.305C>A	p.Ala102Glu		3 of 10	Likely oncogenic*

*These variants were initially classified as somatic variants of uncertain significance using the consensus recommendations for oncogenicity but would qualify as likely pathogenic by germline standards; thus, we would consider these variants likely oncogenic. RBR, RNA-binding region; SNF2, DNA-dependent ATPase domain of the Sucrose Non-Fermenting 2 family.

### Biochemical evaluation

Biochemical evaluation was based on data sent to and performed at the NIH, which included plasma normetanephrine, norepinephrine, metanephrine, epinephrine, 3-methoxytyramine, dopamine, and chromogranin A. Response to systemic therapies was determined by comparing the last biochemical value pre- and post-treatment.

Biochemical values were compiled throughout patients’ metastatic disease course. Given inconsistencies in laboratory reported reference ranges, fold changes above the upper reference limit (URL) were reported. If the patient’s plasma metanephrine amounted to less than 5% of their total metanephrine, normetanephrine, and 3-methoxytyramine sum, corrected by their respective upper reference limits, they were described as having a noradrenergic biochemical phenotype (27).

### Imaging

Per-lesion and per-patient analyses were performed using the following scans available at the NIH: ^18^F-FDOPA PET or PET/CT, ^18^F-FDA PET or PET/CT, ^68^Ga-DOTATATE PET/CT, ^18^F-FDG PET/CT, and ^123^I-MIBG scintigraphy. The duration between functional imaging scans in all patients was less than 3 months except in patient 2, where ^18^F-FDA was performed about a year prior to other scans. Histologic proof of all metastatic lesions was not feasible. Therefore, a composite of all the imaging studies served as a reference standard for the calculation of detection rates. A lesion was classified as true positive if found to be positive on at least two functional imaging modalities (^18^F-FDOPA, ^18^F-FDG, ^68^Ga-DOTATATE, ^18^F-FDA, and ^123^I-MIBG). A positive lesion found on one functional imaging modality and negative on all others was considered a false-positive (**Table 2**). A patient was considered positive regardless of the number of positive lesions present; counting of metastatic lesions was limited to a maximum of 15 lesions per region (lymph nodes, lungs, mediastinum, liver, and abdomen). Ratios were defined by the number of lesions detected by functional imaging compared to the composite reference standard. McNemar’s test was used to determine significance (p-value <0.05) between ^18^F-FDOPA and other imaging modalities as it detected all the lesions on imaging comparator.

**Table 2:** Per-lesion and per-patient detection rates of various imaging modalities.

Detection rates	^18^F-FDOPA PET or PET/CT	^18^F-FDA PET or PET/CT	^68^Ga-DOTATATE PET/CT	^18^F-FDG PET/CT	^123^I-MIBG scintigraphy
**Per-lesion**	81/81 (100, 95.6–100)	45/50 (90.0, 78.2–96.7, p=0.055)	62/75 (82.7, 72.2–90.4, p=0.0001)	40/75 (53.3, 41.5–65.0, p=0.0001)	30/38 (78.9, 62.7–90.5, p=0.0005)
**Per-patient**	4/4 (100, 39.8–100)	3/3 (100, 29.2–100)	3/3 (100, 29.2–100)	3/3 (100, 29.2–100)	2/2 (100, 15.8–100)

Values are expressed in ratios, defined as the number of lesions or patients detected by the imaging modality compared to the total number of lesions or patients evaluated by that modality, followed by the percentages along with 95% confidence intervals in parentheses. McNamar’s test was used to determine significance, and P values are shown between ^18^F-FDOPA and the respective imaging modalities. The number of lesions were limited to 15 per body region. The per-lesion detection rate of ^18^F-FDOPA was statistically superior to all imaging modalities except ^18^F-FDA.

### Immunohistochemistry

Immunohistochemistry (IHC) for ATRX was performed in tumor specimens from the four patients to assess functional correlation of the protein. A Sigma-Aldrich anti-ATRX antibody (polyclonal, HPA001906, St. Louis, MO) was used at a dilution of 1:200 on unstained slides from formalin fixed paraffin embedded (FFPE) tissue using the Ventana Benchmark XT (Ventana, Tucson, AZ, USA). A Santa Cruz Biotechnology anti-FH antibody (monoclonal, sc-100743, Dallas TX) was used at a dilution of 1:100 on unstained slides from formalin fixed paraffin embedded (FFPE) tissue using the Ventana Benchmark XT (Ventana, Tucson, AZ, USA). Validation of the ATRX staining was performed on the daily clinical laboratory control, the same day these patient tumor samples were stained, by the surgical pathologist on clinical service at the Laboratory of Pathology, National Cancer Institute.

## Results

### Patient characteristics

Patient characteristics are summarized in **Supplementary Table 1**. Four male PCC patients had somatic *ATRX* (NM_000489.6) variants detected in primary (patient 3) and metastatic lesions (patients 1, 2, and 4). Patients 2 and 4 had additional somatic *VHL* (NM_000551.4) and *FH* (NM_000143.4) pathogenic variants, respectively, revealed years after their initial diagnosis when comprehensive genetic analysis was re-initiated at the NIH. Age at initial diagnosis ranged from 28 to 66 years (median=52.5 years). PCC sizes ranged from 8.7 to 14 cm (median=12 cm). Two patients (50%, patients 1 and 4) had left PCC, and the remaining (50%, patients 2 and 3) had right PCC. All patients developed metastatic disease.

### Metastatic disease

Clinical characteristics of primary and metastatic disease are summarized in **Supplementary Table 1**. Initially, all patients underwent surgical resection of their PCCs. One patient (25%, patient 3) presented with synchronous metastases, in which PCC and distant metastases were found within 6 months, and the remaining three had metachronous metastases (75%, patients 1, 2, and 4). Time between primary resection and development of metastasis in these three patients ranged from 4–26 years (median=9 years). Two patients (50%, patients 2 and 4) had recurrence in their primary adrenalectomy bed ranging between 9–28 years (median=18.5 years). Patient 2 had recurrence of a C2 paraspinal mass 2 years after resection. This patient, the youngest in our cohort, exhibited the most prolonged interval from primary surgical resection to metastatic disease on imaging. However, it is noteworthy that the patient did not adhere to regular follow-up throughout those 26 years.

All patients presented with multiple metastatic lesions to bones, lungs, and lymph nodes. Three patients (75%, patients 2, 3, and 4) had metastases to the liver. Patient 4 (25%) had metastasis to the mesocolon. Those with pulmonary, hepatic, and osseous disease had multiple metastatic lesions in these locations. Two patients (50%, patients 1 and 2) presented with synchronous organ and osseous metastases, while these were metachronous for the remaining two (50%, patients 3 and 4).

### Genetics

Three of four somatic *ATRX* variants were classified as likely oncogenic. Patients 1 and 4 both had missense variants in exon 20: c.5248C>G (p.Pro1750Ala) and c.5229G>T (p.Arg1743Ser), respectively, both in the ATP-binding SNF2 protein domain (20). REVEL scores for these variants in patients 1 and 4 were 0.94 and 0.709, respectively, providing “strong” and “supporting” levels of computational evidence, respectively, of pathogenicity for these variants (28). Given that the germline classification of the *ATRX* variant for patient 1 would be considered likely pathogenic, this variant was considered likely oncogenic in the somatic context (25, 29). Of all these somatic *ATRX* variants, only this one (p.Pro1750Ala) had been reported previously in the Catalogue of Somatic Mutations in Cancer (COSMIC: https://cancer.sanger.ac.uk/cosmic), found in a uterine leiomyosarcoma. Patients 2 and 3 had frameshift truncating variants in exon 9: c.3230del (p.Ser1077del) and c.2018dup (p.Thr674fs), respectively, the latter of which is located in the RBR domain (20). Both of these variants met criteria to be classified as likely oncogenic (**Supplementary Table 2**).

In addition to each hemizygous somatic *ATRX* oncogenic variant, two patients (2 and 4) were found to have additional somatic variants: *VHL* c.505C>G (p.Leu169Val) and *FH* c.305C>A (p.Ala102Glu), respectively, both of which were classified as likely oncogenic missense variants (**Table 1**; **Supplementary Table 2**). None of these variants (in *ATRX*, *VHL*, or *FH*) were present in ClinVar.

### Immunohistochemistry

**Figure 2:**
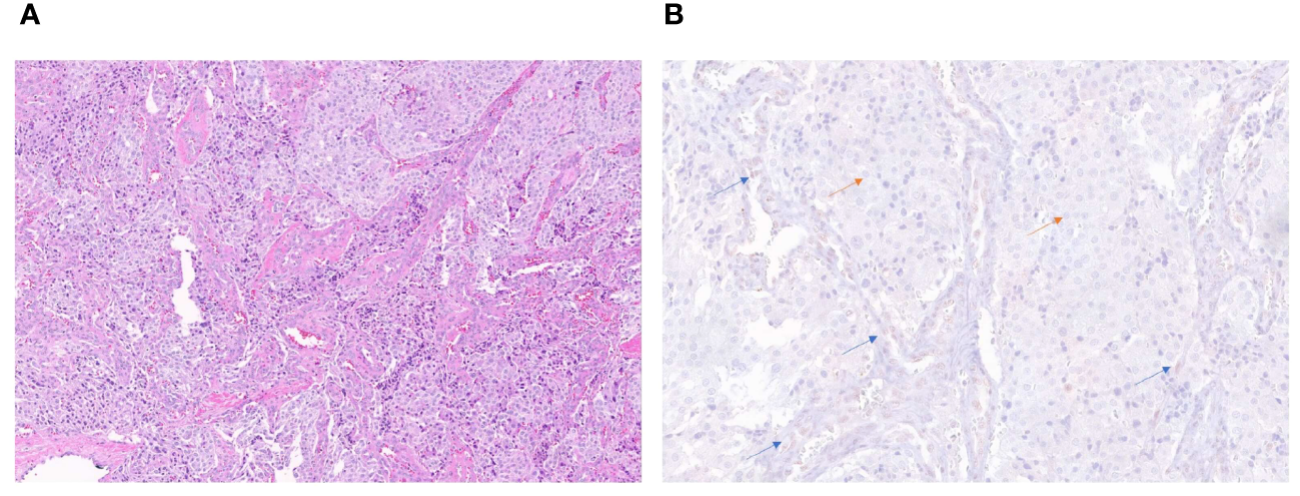
H&E stain (20x) of the periaortic lymph node metastatic lesion in patient 4 **(A)**. Immunohistochemistry stain (40x) showing retained ATRX protein (brown staining) in internal control of endothelial and stromal cells of vessels (blue arrows) and loss of protein in tumor cells (orange arrows) **(B)**.

ATRX staining for patient 4 tumor sample showed loss of nuclear ATRX in the tumor samples with faintly positive stromal cells serving as an internal control (**Figure 2**). ATRX staining in tumor samples from patients 2 and 3 failed to display an adequate internal control staining in the stromal cell nuclei, and the tumor sample from patient 1 showed subsets of tumor cell nuclei with ATRX loss and ATRX retention. Samples from patients 1, 2, and 3 were determined to be inadequate for interpretation due to these possible technical concerns in the staining pattern. Loss of FH protein in the tumor cells was confirmed by IHC (**Supplementary Figure 1**) in patient 4, with validation performed on the same day clinical laboratory control sample.

### Biochemical phenotypes

All patients underwent biochemical analysis, as described above (**Supplementary Table 3**). However, assessment was not available at regular intervals since patients were largely managed and diagnosed outside the NIH.

#### Initial presentation

Out of four patients, two (patients 1 and 3) had biochemical evaluation at initial diagnosis. Patient 1 had plasma normetanephrine elevation 15.3-fold above the URL. Patient 3 (with synchronous metastases) had elevated plasma normetanephrine, norepinephrine, metanephrine, epinephrine, and dopamine by 64.9-, 1.8-, 17.9-, 5.9-, and 14.6-fold above the URL, respectively.

#### Metastatic disease

Biochemistries were followed after metastatic disease was detected. All patients had plasma elevations in normetanephrine (median=1.3-fold above the URL, 0.02–64.9-fold) and norepinephrine (median=1.1-fold above the URL, 0.02–41.9-fold). Additionally, all patients had plasma 3-methoxytyramine elevations (median=4.5-fold above the URL, 1.1–71.8-fold). Three patients (75%, patients 2, 3, and 4) had plasma dopamine elevation (median=0.52-fold above the URL, 0.04–25.5-fold). Two patients (50%, patients 2 and 3) had metanephrine (median = 0.3-fold above the URL, 0.05–18.0-fold) and epinephrine elevations (median = 3.0-fold above the URL, 0.1–5.9-fold). Additionally, all had significant CgA elevation (median= 29.1-fold above the URL, 1.0–204.9-fold).

In summary, at the time of metastatic involvement, all had a noradrenergic biochemical phenotype with significant plasma 3-methoxytyramine elevation, and CgA elevation.

### Anatomical and functional imaging studies

#### Primary tumors

Multiple modalities of anatomical and functional imaging were used to diagnose primary adrenal tumors in our patient cohort. The functional imaging studies are summarized in **Supplementary Table 4**. Detection was performed by abdominal ultrasound followed by CT in three patients (75%, patient 1, 3, and 4), and MRI in one patient (25%, patient 2).

#### Metastatic tumors

Functional imaging studies evaluating metastatic disease are summarized in **Supplementary Table 4**. Of four patients, all (100%) underwent ^18^F-FDOPA PET (n=1, 25%, patient 1) or PET/CT (n=3, 75%; patients 2–4), and three (75%, patients 2–4) underwent ^68^Ga-DOTATATE and ^18^F-FDG PET/CT. Three patients (75%) had ^18^F-FDA PET (n=1, 33%, patient 1) or PET/CT (n=2, 67%, patients 2 and 4). Finally, two patients had additional ^123^I-MIBG imaging (50%, patients 2 and 3). ^68^Ga-DOTATATE PET/CT did not detect liver metastases in patient 3, which were seen on CT, ^123^I-MIBG scintigraphy, ^18^F-FDG, and ^18^F- FDOPA PET/CT.

The total number of lesions identified among all patients was 81. The per-lesion detection rate of ^18^F-FDOPA PET or PET/CT was 100% (81/81, 95% CI 95.6–100%), which was superior compared to that of ^68^Ga-DOTATATE PET/CT (82.7% [62/75], 95% CI 72.2–90.4%, p=0.0001), ^18^F-FDG PET/CT (53.3% [40/75], 95% CI 41.5–65.0%, p=0.0001), and ^123^I-MIBG scintigraphy (78.9% [30/38], 95% CI 62.7–90.5%, p=0.0005). The difference in per-lesion detection rates between ^18^F-FDOPA PET/CT and other functional imaging modalities reached statistical significance except for ^18^F-FDA PET or PET/CT (90% [45/50], 95% CI 78.2–96.7%, p=0.055), which tended towards significance (p=0.055). ^18^F-FDG PET/CT performed the worst in comparison to all other functional imaging modalities. The per-patient detection rate of all performed functional imaging modalities was 100% (**Table 2**).

### Treatment outcomes

Three patients (patients 1, 3, and 4) have succumbed to metastatic disease. Overall survival from the time of diagnosis ranged from 2 to 26 years with a median of 11 years. One patient (25%, patient 2) is alive 36 years after initial diagnosis with stable disease 2 years after completion of 8 cycles of Lutathera^®^.

Patients 1, 2, and 4 received various therapies, including cytotoxic chemotherapy, targeted radiotherapies, external beam radiation, and somatostatin analogs. Patient 1 received ^131^I-MIBG therapy, with mixed response, specifically, shrinkage in one lymph node and progression in pulmonary disease, ultimately requiring surgical pulmonary debulking. Upon continued progression and widespread metastatic disease, he received 3 cycles of CVD with progression on therapy leading to death from metastatic disease.

Patient 2 received IMRT (5400 cGy) to the right sacral area with shrinkage and was also treated with Sandostatin^®^ LAR and sunitinib for widespread organ metastases, during which progression was noted. He subsequently received four cycles of Lutathera^®^, leading to shrinkage of liver, lung, and lymph node lesions, along with stabilization of other areas for 3 years. Once progression was noted, he restarted Lutathera^®^ for an additional 4 cycles and continues to have stable disease 2 years after treatment.

Lastly, patient 4 received 4 cycles of Lutathera^®^, followed by lanreotide and CVD, with progression, leading to death from metastatic disease.

Overall, treatment with cytotoxic chemotherapy and a tyrosine kinase inhibitor (TKI) were not effective in stabilizing or shrinking tumors. Systemic radiation with ^131^I-MIBG and IMRT targeted to sacral metastasis showed response in a periaortic and multiple abdominal lymph nodes (patient 1, 25%) and a sacral osseous metastasis (patient 2, 25%), respectively. Lanreotide treatment in two patients (patients 2 and 4) showed the most durable response with stabilization and shrinkage of tumors in patient 2.

## Discussion

Some reports have described somatic *ATRX* variants as clinically important risk factors for aggressive/metastatic PCCs/PGLs (23, 24), however, few have described their detailed associations with biochemical phenotypes, functional imaging, or treatment outcomes and survival of patients with these tumors. We identified four patients with somatic *ATRX* variants and metastatic PCC. Patients 2 and 4 had additional somatic pathogenic variants in *VHL* and *FH*, respectively, which are known PCC/PGL susceptibility genes. All four patients were males, who could exhibit greater susceptibility to effects of *ATRX* loss-of-function variants due to hemizygosity, but based on this small series, we cannot definitively infer biological outcomes based on sex differences. Further investigation should be performed in a greater subset of patients, including females with PCC/PGL found to have oncogenic *ATRX* variants.

This mini-case series corroborates previous evidence that presence of somatic *ATRX* variants is associated with aggressive/metastatic PCC. In our cohort, three out of four patients (patient 1, 3, and 4) have succumbed to metastatic disease. Patient 4 harbored a somatic *FH* pathogenic variant, which is often associated with metastatic disease (1). However, prior seminal studies have shown aggressive/metastatic behavior in the presence of *ATRX* variants, even in the presence of other well-known aggressive pathogenic variants. For example, Fishbein et al. showed that approximately 67% of their PCC/PGL samples with somatic *ATRX* pathogenic variants, also harbored *SDHB* germline pathogenic variants. Telomere FISH revealed that all had ALT activation, and clinically aggressive behavior (23). The aggressive/metastatic nature of PCC with somatic *ATRX* pathogenic variants suggests a need for heightened frequency of clinical evaluation and development of specific recommendations for patients harboring these variants. Additionally, use of telomere FISH to assess for ALT as a result of oncogenic *ATRX* variants may be helpful in clinical assessment in these patients when observing tumor specimens by histopathology as was done in Fishbein et al. (23), but additional studies are needed to validate its clinical utility.

To date, biochemical phenotypes associated with *ATRX* variants have not been well reported. All four patients presented with a noradrenergic biochemical phenotype with significant elevations in 3-methoxytyramine, indicating substantial dopamine production. Interestingly, CgA was also substantially elevated in all patients, especially with metastatic disease. Patients 2 and 4 harbored variants in PCC/PGL cluster 1 genes, *VHL* and *FH*, respectively, which have been known to express the noradrenergic biochemical phenotype (30). Thus, while we cannot definitively assert that *ATRX* variants are driving the noradrenergic phenotype, tumors with somatic *ATRX* variants in this study were associated with this phenotype and with elevation in 3-methoxytyramine. Future analysis in a larger cohort of patients should be performed to further uncover the impact of *ATRX* variants on biochemical phenotype.

Our small cohort of PCCs with *ATRX* variants also provides insight on functional imaging modalities in these patients. ^18^F-FDOPA PET/CT was shown to be superior to all other functional imaging modalities, with 100% lesion detection in each patient as compared to the reference standard. ^18^F-FDG PET/CT performed the worst in these patients, with a per-lesion detection of only 53.3%. Thus, ^18^F-FDOPA PET/CT seems to be the imaging modality of choice for these patients, similar to patients having pathogenic variants in cluster 1b or 2 genes or those with sporadic PCCs (31–35). It is important to note that while patient 2, with a pathogenic *VHL* variant, already falls into cluster 1b, associated with preferred ^18^F-FDOPA PET/CT imaging, patient 4, with an *FH* variant (cluster 1a) may have been appropriately imaged with ^68^Ga-DOTATATE PET/CT or ^18^F-FDOPA PET/CT given prior reports (36, 37). These data suggest that the presence of *ATRX* variants favors ^18^F-FDOPA PET/CT as the imaging modality of choice.

While PCCs/PGLs are highly heritable tumors that show considerable heterogeneity (38), none of these patients were found to have germline variants in PCC/PGL susceptibility genes. A substantial proportion of these tumors are also due to somatic variants in PCC/PGL susceptibility genes, including many of the same genes implicated in heritable disease (*NF1, SDHx*, *VHL*, and *RET*), but also somatic (or somatic mosaic) variants in genes, including *HRAS*, *FGFR1*, *EPAS1*, *H3F3A*, and *ATRX* (39, 40). As our understanding of the molecular biology of these tumors improves, knowledge of the underlying genetic pathogenesis may inform molecular targeted therapies, as demonstrated in a patient with an *EPAS1* gain-of-function mutation (41). Therefore, genetic testing for both germline as well as somatic etiologies is crucial for these tumors, as suggested by other experts previously (42). Additionally, multi-omic comparisons between primary and metastatic sites in patients may be useful in forming a deeper understanding of the fundamental biology of tumor evolution in future cohorts.

The five-year survival for those with metastatic PCC/PGL is 37%, and male biological sex and presence of synchronous metastases are associated with higher mortality (43), even though median overall survival is approximately 116.0 months (44). In our cohort, two patients presented with synchronous metastases. Most presented with large PCC (median=12 cm), and therefore, such large tumors could further contribute to metastatic disease as described previously (2, 43). PCC is a tumor type known to have better clinical outcomes (45), however our PCC patients developed metastatic disease and ultimately died, thus suggesting that the presence of *ATRX* variants and perhaps a large tumor size, rather than the primary location, contributed to adverse outcomes. While two patients had additional pathogenic *VHL* or *FH* variants, these have been described to have a lesser impact on metastatic disease (1, 46). However, since all patients with *ATRX* variants presented with aggressive/metastatic disease despite initially presenting with PCC (low metastatic risk) and *VHL or FH* variants (low-moderate metastatic risk), we speculate, as others, that *ATRX* variants contribute to metastatic risk (23). Future prospective studies with a larger cohort of patients could deepen our understanding of the nature of *ATRX* variants in PCC/PGL when compared to appropriately matched controls without these variants.

Therapeutic outcomes cannot be definitively established in this small study; however, it seems that these patients do not respond to CVD or TKIs but could respond (at least partially) to somatostatin analogs and systemic radiotherapy using either ^131^I-MIBG (Azedra^®^) or ^177^Lu-DOTATATE (Lutathera^®^), based on relative avidities of metastatic lesions for ^123^I-MIBG scintigraphy or ^68^Ga-DOTATATE, respectively (47–49). In summary, here we present four male PCC patients with somatic *ATRX* variants presenting with metastatic/aggressive disease. While this case series cannot come to definite conclusions due to its limited size, we found that these patients presented mainly with a noradrenergic biochemical phenotype, elevated 3-methoxytyramine levels, and large tumors. ^18^F-FDOPA PET/CT performed best on per-lesion detection, and systemic radiotherapy using ^131^I-MIBG (Azedra^®^) or ^177^Lu-DOTATATE (Lutathera^®^) may be considered as a form of therapy in these patients with aggressive/metastatic disease, as they led to limited therapeutic response and stabilization of disease compared to other treatment options.

## Data Availability

Original datasets from the patients are available in the publicly accessible repository dbGaP with accession number phs002405.v2.p1.

## Author disclaimer

The Authors’ (KP, MK, AJ, MP, JC, TP, KC, ST, AD, FL, DT, JR) contribution to the Work was done as part of the Authors’ official duties as NIH employees and is a Work of the United States Government. Therefore, copyright may not be established in the United States, 17 U.S.C. § 105. If the Publisher intends to disseminate the Work outside of the U.S., the Publisher may secure copyright to the extent authorized under the domestic laws of the relevant country, subject to a paid-up, nonexclusive, irrevocable worldwide license to the United States in such copyrighted work to reproduce, prepare derivative works, distribute copies to the public and perform publicly and display publicly the work, and to permit others to do so.
