# Correction: Case series: *ATRX* variants in four patients with metastatic pheochromocytoma

**DOI:** 10.3389/fendo.2025.1736207

**Published:** 2025-11-14

**Authors:** Briana N. Cortez, Mickey J. M. Kuo, Abhishek Jha, Mayank Patel, Jorge A. Carrasquillo, Tamara Prodanov, Kailah M. Charles, Sara Talvacchio, Alberta Derkyi, Frank I. Lin, David Taïeb, Jaydira Del Rivero, Karel Pacak

**Affiliations:** 1 Section on Medical Neuroendocrinology, Eunice Kennedy Shriver National Institute of Child Health and Human Development, National Institutes of Health, Bethesda, MD, United States; 2 Medical Genetics Branch, National Human Genome Research Institute, National Institutes of Health, Bethesda, MD, United States; 3 Center for Cancer Research, Laboratory of Pathology, National Cancer Institute, Bethesda, MD, United States; 4 Molecular Imaging Branch, National Cancer Institute, National Institutes of Health, Bethesda, MD, United States; 5 Department of Nuclear Medicine, La Timone University Hospital & Centre de Recherches en Cancérologie de Marseille (CERIMED) & French Institute of Health and Medical Research (Inserm) UMR1068 Marseille Cancerology Research Center, Institut Paoli-Calmettes, Aix-Marseille University, Marseille, France; 6 Developemental Therapeutics Branch, National Cancer Institute, National Institutes of Health, Bethesda, MD, United States

**Keywords:** case report, pheochromocytoma, ATRX, prognosis, imaging

The **Conflict of interest** statement was erroneously given as “The authors declare that the research was conducted in the absence of any commercial or financial relationships that could be constructed as a potential conflict of interest”. The correct **Conflict of interest** statement is “DT is a founder, stakeholder, and the Chief Medical Officer of SilonTx, a company with interest in molecular medicine and nuclear medicine for cancer and rare diseases. The remaining authors declare that the research was conducted in the absence of any commercial or financial relationships that could be construed as a potential conflict of interest. The author(s) declared that they were an editorial board member of Frontiers, at the time of submission. This had no impact on the peer review process and the final decision.”

The original version of this article has been updated.

