# Supplementary material for: Case Series: ATRX Variants in Four Patients with Metastatic Pheochromocytoma
Source: Front Endocrinol (Lausanne). 2024 Sep 16;15:1399847. doi: 10.3389/fendo.2024.1399847 (PMC11439680; doi:10.3389/fendo.2024.1399847)
Supplement: Supplementary file 1 [file Presentation1.pdf]

	Gender, Age at initial diagnosis (years)	Follow-up at the NIH (years)	Genetic Variants	Biochemical Phenotype	Presenting Signs and Symptoms	Primary Tumor Location	Primary Tumor Size	Metastatic Regions	Surgery	Therapy	Positive Functional Imaging	Outcome
1	Male, 60	5	*ATRX* (c.5248C>G, p.Pro1750Ala)	Noradrenergic	HTN, diaphoresis, palpitations	Left adrenal gland	14 cm	Lungs<br>Lymph nodes<br>Bone	Left adrenalectomy/nephrectomy (2001)<br><br>Right middle lobectomy and mediastinal node dissection (2008)	^{131}I-MIBG (2005)<br><br>cyclophosphamide, vincristine, dacarbazine (3 cycles, 2010)	^{123}I-MIBG scintigraphy<br>^{18}F-FDG PET/CT<br>^{18}F-FDA PET<br>^{18}F-FDOPA PET/CT<br>Technetium-99m/MDP bone scan	Deceased, due to metastatic disease
2	Male, 28	5	*ATRX* (c.3230del, p.Ser1077del)<br><br>*VHL* (c.505C>G, p.Leu169Val)	Noradrenergic	HTN, pounding headaches, abdominal pain, nausea	Right adrenal gland	13 cm	Recurrence in right adrenal surgical bed<br>Recurrence in C2 lamina<br>Lungs<br>Liver<br>Lymph nodes<br>Bone	Right adrenalectomy (1978)<br><br>Exploratory laparotomy, open cholecystectomy and resection of the right aortocaval mass (2014)<br><br>Resection of C2 paraspinal mass (2015)	IMRT 5400 cGy to the right sacrum (2015)<br><br>Sandostatin® (2016)<br><br>Sunitinib (2017)<br><br>Lutathera® (2018, 2021)	^{123}I-MIBG scintigraphy<br>^{18}F-FDG PET/CT<br>^{18}F-FDA PET/CT<br>^{18}F-FDOPA PET/CT<br>^{68}Ga-DOTATATE PET/CT	Living with stable metastatic disease as monitored by imaging
3	Male, 66	4	*ATRX* (c.2018dup, p.Thr674fs)	Noradrenergic	HTN, right sided back pain, pounding headache, vertigo, fatigue, multiple subcutaneous tumors	Right adrenal gland	8.7 cm	Lungs<br>Liver<br>Bone	Laparoscopic right adrenalectomy (2019)	None	^{123}I-MIBG scintigraphy<br>^{18}F-FDG PET/CT<br>^{18}F-FDOPA PET/CT<br>^{68}Ga-DOTATATE PET/CT	Deceased, due to metastatic disease
4	Male, 45	9	*ATRX* (c.5229G>T, p.Arg1743Ser)<br><br>*FH* (c.305C>A, p.Ala102Glu)	Noradrenergic	HTN, palpations, flushing, uncontrolled type 2 diabetes mellitus with hyperglycemic episodes	Left adrenal gland	11 cm	Recurrence in left adrenal bed<br>Lungs<br>Mediastinum<br>Liver<br>Mesocolon<br>Peritoneum<br>Lymph nodes<br>Bone	Left adrenalectomy/nephrectomy (2000)<br><br>Resection of metastasis at the left adrenal bed, left subdiaphragmatic region, and left mesocolon (2009)<br><br>Exploratory laparotomy, lysis of adhesions, resection of left distal periaortic mass, left periaortic mass at the level of the adrenal bed, and retropancreatic mass (2014)	Lutathera® (2018)<br><br>Lanreotide (2018)	^{123}I-MIBG scintigraphy<br>^{18}F-FDG PET/CT<br>^{18}F-FDA PET/CT<br>^{18}F-FDOPA PET/CT<br>^{68}Ga-DOTATATE PET/CT<br>Technetium-99m/MDP bone scan	Deceased, due to metastatic disease

Supplementary Table 1. Clinical characteristics of four PCC patients with somatic *ATRX* variants.

Patient ID	Tissue	Germline or Somatic	Gene	Variant (c.)	Variant (p.)	Oncogenicity Classification	Oncogenicity Score	Oncogenicity Evidence	Pathogenicity Classification	REVEL	VAF
1	Right middle lobe pulmonary nodule	Somatic	*ATRX*	c.5248C>G	p.Pro1750Ala	Uncertain significance	2	OP1, OP4	Likely pathogenic	0.94	0.5
2	Right retrocaval mass	Somatic	*ATRX*	c.3230del	p.Ser1077del	Likely oncogenic	9	OVS1, OP4	N/A	N/A	0.684
			VHL	c.505C>G	p.Leu169Val	Likely oncogenic	6	OM1, OP1, OP4	N/A	0.774	0.227
3	Right adrenal PCC	Somatic	*ATRX*	c.2018dup	p.Thr674fs	Likely oncogenic	9	OVS1, OP4	N/A	N/A	0.75
4	Left adrenal surgical bed mass	Somatic	*ATRX*	c.5229G>T	p.Arg174Ser	Uncertain significance	2	OP1, OP4	Uncertain significance	0.709	0.723
			FH	c.305C>A	p.Ala102Glu	Uncertain significance	2	OP1, OP4	Likely pathogenic	0.941	0.687

Supplemental Table 2. Expanded form of table 3 showing the evidence used to define oncogenicity of somatic variants based on oncogenicity scores (25). Pathogenicity classifications were performed for variants interpreted as variants of uncertain significance based on somatic oncogenicity scores and were obtained using the 2015 ACMG guideline using REVEL scores (26, 28, 29). REVEL, rare exome variant ensemble learner; VAF, variant allele frequency (in tumor tissue).

		Plasma Normetanephrine (Median, Range)	Plasma Norepinephrine (Median, Range)	Plasma Metanephrine (Median, Range)	Plasma Epinephrine (Median, Range)	Plasma 3-methoxytyramine (Median, Range)	Plasma Dopamine (Median, Range)	Plasma Chromogranin A (Median, Range)
Patient 1	Primary evaluation	Elevated (15.3x URL)	NA	NA	NA	NA	NA	NA
	Metastatic disease	Elevated (2.1x, 1.2-61.3)	Elevated (-0.5x, -0.6-1.6)	Normal	Normal	Elevated (24.5x URL)	Normal	Elevated (2.3x, 1.8-40.8)
	Change after ^{131}I-MIBG therapy	Decreased by 0.7x	Decreased by 1.5x, normalized	Normal	Normal	NA	Normal	Decreased by 0.1x
Patient 2	Primary evaluation	NA	NA	NA	NA	NA	NA	NA
	Metastatic disease	Elevated (26.1x, 0.1-72.9)	Elevated (0.9x, -0.5-3.8)	Elevated (-0.6, -0.6-1.4)	Elevated (-0.6x, -0.8-0.1)	Elevated (4.7x, 1.1-71.8)	Elevated (0x, -0.2-1.1)	Elevated (35.1, 1.7-204.9)
	Change after Lutathera® (Cycles 1-4)	Decreased by 5.3x	Decreased by 4.0x, normalized	Increased by 0.3x	Normal	NA	Decreased by 1.1x	Decreased by 6.9x
	Change after Lutathera® (Cycles 5-8)	Decreased by 0.6x	Decreased by 0.8x	Normal	Normal	NA	Normal	Decreased by 2.5x
Patient 3	Primary evaluation	Elevated (64.9x URL)	Elevated (1.8x URL)	Elevated (17.9x URL)	Elevated (5.9x URL)	NA	Elevated (14.6x URL)	Elevated (51.7x URL)
	Metastatic disease	Elevated (31.5x, 5.9-64.9)	Elevated (0.9x, -0.04-1.8)	Elevated (9.2x, -0.01, 21.1)	Elevated (2.6x, -0.6-5.9)	Elevated (1.4x URL)	Elevated (8.2x,1.8-14.6)	Elevated (32.3x, 12.8-51.7)
Patient 4	Primary evaluation	NA	NA	NA	NA	NA	NA	NA
	Metastatic disease	Elevated (25.2x, 1.4-138.4)	Elevated (4.9x, 0.7-41.9)	Normal	Normal	Elevated (4.4x URL)	Elevated (0.3, -0.5, 25.5)	Elevated (10.2x, 1-66.4)
	Change after Lutathera® (Cycles 1-4)	Decreased by 1.7x	NA	Normal	NA	NA	NA	Decreased by 0.9x
	Change after Lanreotide	Decreased by 1.3x	NA	Normal	NA	NA	NA	Decreased by 0.7x

Supplementary Table 3. Biochemical trends at primary evaluation, throughout metastatic disease course, and changes after systemic treatments are reported. Those whose values remained normal prior to and after treatment were noted as “Normal.” In parentheses, the values are reported as fold changes above the upper reference limit. Median and range are reported for those patients who had multiple biochemical values throughout their metastatic disease duration. NA, not available; URL, upper reference limit.

		^{123}I-MIBG scintigraphy	^{18}F-FDG PET/CT	^{18}F-FDOPA PET or PET/CT	^{18}F-FDA PET or PET/CT	^{68}Ga-DOTATATE PET/CT
Patient 1	Adrenal Tumor (Primary)					
	Lungs			+	+	
	Lymph Nodes			+	+	
	Bone			+	+	
	Mediastinum			+	+	
Patient 2	Adrenal Tumor (Primary)					
	Adrenal Tumor (Recurrence)			+		+
	Lungs	-	-	+	-	+
	Lymph Nodes	-	-	+	-	+
	Bone	-	+	+	+	+
Patient 3	Adrenal Tumor (Primary)		+	+		+
	Lungs	+	+	+		+
	Liver	+	+	+		-
	Bone	+	+	+		+
Patient 4	Adrenal Tumor (Primary)					
	Adrenal Tumor (Recurrence)	+	+			
	Mediastinum		+	+	+	+
	Lungs		+	+	+	+
	Liver		+	+	+	+
	Lymph Nodes		+	+	+	+

Supplemental Table 4. Lesion detection per functional imaging modality in evaluation of primary, recurrent, and metastatic disease. Presence of disease is noted as (+), absence of disease is noted as (-), and scan not available or not performed are left blank.

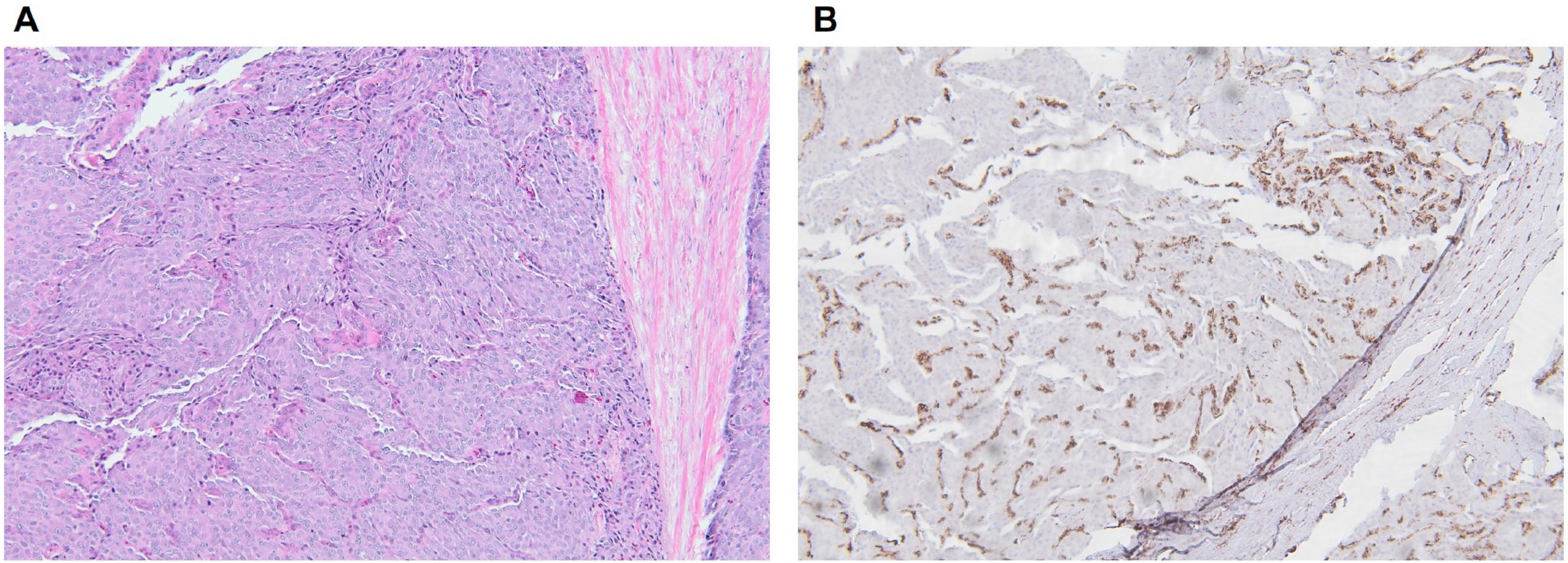

Supplemental Figure 1: H&E stain (20x) of the periaortic lymph node metastatic lesion in patient 4 (**A**). Immunohistochemistry staining (40x) showing loss of FH protein in clusters of tumor cells (**B**).
